# Identifying Object Categories from Event-Related EEG: Toward Decoding of Conceptual Representations

**DOI:** 10.1371/journal.pone.0014465

**Published:** 2010-12-30

**Authors:** Irina Simanova, Marcel van Gerven, Robert Oostenveld, Peter Hagoort

**Affiliations:** 1 Max Planck Institute for Psycholinguistics, Nijmegen, The Netherlands; 2 Donders Institute for Brain, Cognition and Behaviour, Radboud University Nijmegen, Nijmegen, The Netherlands; 3 Institute for Computing and Information Sciences, Radboud University Nijmegen, Nijmegen, The Netherlands; University of Leuven, Belgium

## Abstract

Multivariate pattern analysis is a technique that allows the decoding of conceptual information such as the semantic category of a perceived object from neuroimaging data. Impressive single-trial classification results have been reported in studies that used fMRI. Here, we investigate the possibility to identify conceptual representations from event-related EEG based on the presentation of an object in different modalities: its spoken name, its visual representation and its written name. We used Bayesian logistic regression with a multivariate Laplace prior for classification. Marked differences in classification performance were observed for the tested modalities. Highest accuracies (89% correctly classified trials) were attained when classifying object drawings. In auditory and orthographical modalities, results were lower though still significant for some subjects. The employed classification method allowed for a precise temporal localization of the features that contributed to the performance of the classifier for three modalities. These findings could help to further understand the mechanisms underlying conceptual representations. The study also provides a first step towards the use of concept decoding in the context of real-time brain-computer interface applications.

## Introduction

Identification of the neural processes underlying semantic representations is a key challenge in cognitive neuroscience. Different hypotheses have been proposed on how representations of particular concepts establish a system of conceptual knowledge. The general consensus is that shared object properties are reflected in the organization of the semantic system and that the system generalizes across concepts that belong to a particular category (such a *animals*, *tools*, or *buildings*). The notion of category specificity in the organization of object knowledge emerged in the 1980s, when Warrington and colleagues first reported on patients with selective impairment for one semantic category compared to other semantic categories [1]–[3]. Since those initial investigations, a large number of studies have confirmed the phenomenon of category–specific semantic deficits. Patients have been reported with impairments for all types of knowledge about a particular category such as, for instance, living things. Such patients are severely impaired for both perceptual (“Does a cow have a mane?”) and functional (“Does a whale fly?”) knowledge of living things, but are within normal range for both types of knowledge for non-animals [4]–[6].

Differences in category-related brain activity have been demonstrated with various neuroimaging methods in healthy subjects, for living things versus manmade objects, and for several specific object categories such as faces, body parts, animals, fruits/vegetables, buildings, tools and furniture (for a recent review see [7]–[9]). Differential activation, suggesting a specific functional organization, has been shown in processing both visual and verbal stimulus modalities. For some types of objects, the functional organization by semantic category has been demonstrated within a given modality, e.g. category–specificity in the visual pathway for faces [10], [11] or for living versus nonliving entities [12]–[15]. It has also been shown that objects and their sensory or functional attributes (such as tool-associated actions) activate the same neural regions [16]–[19], suggesting that these regions are implicitly involved in concept representation.

Modern theories about conceptual representation share the view that the semantic system relates to perceptual and functional attributes of objects that are coded in respective sensory or motor areas. However, there are two broad groups of theories. Theories within the first group assume that each concept is represented as a set of attributes in a distributed system [20], [21]. Concepts from one semantic domain have highly correlated attributes, resulting in category-specific effects. However the semantic system is undifferentiated in the sense that there are no explicit boundaries according to object category, and there is no categorical structure at the level of functional anatomy. Alternatively, theories within the second group assume that a dissociable neural substrate is involved in representing different semantic categories. One such theory, the sensory/functional, initially proposed by Warrington and McCarthy [1], [3] and later modified by others [22], [23], suggests that concepts are essentially grounded in sensory and functional semantic subsystems, and conceptual categories with different sensory or functional emphasis are represented in different subsystems. A second theory within this group, the distributed domain-specific hypothesis [4], [6], [24], suggests that, beyond the sensory and motor properties, there also exist semantic constraints. According to this theory, semantic domains, such as living animals, for which fast and efficient recognition could have had a survival advantage in evolutionary history, have different neuronal substrates. It is suggested that semantic domain is a constraint on the functional organization at both a conceptual level and at the level of visual perception [6].

In recent years, a number of studies have demonstrated the possibility to discriminate retrieval of conceptual categories in functional MRI data, using multivariate analysis methods [25]–[28]. In contrast to conventional univariate methods, multivariate analysis takes into account the full spatial pattern of brain activity. This has been shown to increase sensitivity when analyzing human neuroimaging data [29] and may help to elucidate the nature of semantic representations. The goal of multivariate analysis is to learn a model that best explains the observed data, often quantified in terms of predictive performance (how well does the model predict experimental condition from measured data). Once the model is learned, the obtained parameter estimates can be mapped back to native space, yielding so-called importance maps. These importance maps inform about the relative importance of data features in space and/or time with respect to predicting the experimental condition in single trials. Recently, van Gerven and colleagues introduced a Bayesian approach to multivariate analysis for the interpretation of neuroimaging data [30]. The approach makes it possible to 1) quantify uncertainty about the relative importance of data features and 2) impose constraints on the obtained models based on prior neuroscientific knowledge.

In the current study we applied the Bayesian approach to identify concept-related neuronal activity from event-related brain potentials (ERPs). We presented stimuli of two semantic categories: *animals* and *tools*, and trained a classifier to discriminate these categories. We estimated classification performance and interpret the obtained importance maps at a single-subject level for three stimulus modalities: auditory (an object's spoken name), visual (a drawing of an object), and orthographic (an object's written name). The use of ERPs as the basis for classification was guided by a number of considerations. First, electroencephalography (EEG) has a well-documented ability to characterize certain brain states, in particular the processing of different semantic categories [19], [31]–[36]. Second, the high temporal resolution of EEG allows a fine-grained characterization of concept retrieval in terms of the electrophysiological patterns that make decoding possible. Third, the development of EEG-based semantic-decoding algorithms is interesting from an applications perspective since the temporal resolution of EEG allows decoding in real-time [37]. When it becomes possible to decode conceptual information from EEG, a brain-computer interface system that transforms lexical concepts into a written or spoken output could become a reality.

## Methods

### Participants

Twenty-four native Dutch speakers (10 males and 14 females, 18–28 years of age) participated in the study; four of them were selected as a pilot group (see “Optimization of the analysis”). All participants were right-handed, and reported that they did not suffer from any psychological or neurological disorders. The experiments were approved by the local ethics committee (Commissie Mensgebonden Onderzoek Regio Arnhem-Nijmegen), and all the subjects gave written informed consent prior to the experiment. Subjects received either monetary compensation or course credits for their participation.

### Stimuli

Concepts from three semantic categories were used: two relevant categories (*animals*, *tools*) and a task category that varied across subjects, either *clothing* or *vegetables*. There were four exemplars per category, see Table 1. All exemplars were monosyllabic and were matched for frequency per million (mean±SD = 18.25±9.55) based on CELEX (Max Planck Institute for Psycholinguistics, The Netherlands, 2001).

**Table 1:** Relevant items used in the experiment.

	Orthography	Phonetics
“animals”	koe *(cow)*	ku
	beer *(bear)*	be:r
	leeuw *(lion)*	lew
	aap *(ape)*	a:p
“tools”	bijl *(axe)*	bεil
	schaar *(scissors)*	s÷a:r
	kam *(comb)*	kαm
	pen *(pen)*	pεn

All exemplars were presented in three modalities: auditory (spoken Dutch words recorded digitally at 16 bits with a sampling rate of 44.100 Hz), visual (black line drawings on white background) [38] and orthographical (written Dutch words, black letters on white background). Pictures were matched for familiarity and complexity. Each of the relevant items was repeated eighty times in each modality. Task items were repeated sixteen times and shown approximately once per ten relevant items. The text or picture stimuli were presented for 300 ms and were followed by a blank screen with a random duration between 1000–1200 ms. Subsequently, the next item was presented. The interval between auditory stimuli was also between 1000–1200 ms and a fixation cross was shown on the screen during the auditory presentation.

### Experimental design

All stimuli were presented in twelve blocks with audio, picture and text stimuli in separate blocks. The order of blocks was alternated across subjects. In each run, the same full set of concepts was used and their order was randomized. The experiment lasted about eighty minutes, with a short break between blocks. Participants were instructed to respond upon appearance of items from the classification irrelevant task category (clothing or vegetables). With this procedure participants were forced to categorize the presented items without overtly discriminating between relevant classes. Responses were made by pressing a button with the right hand index finger.

### EEG recording and processing

**Figure 1:**
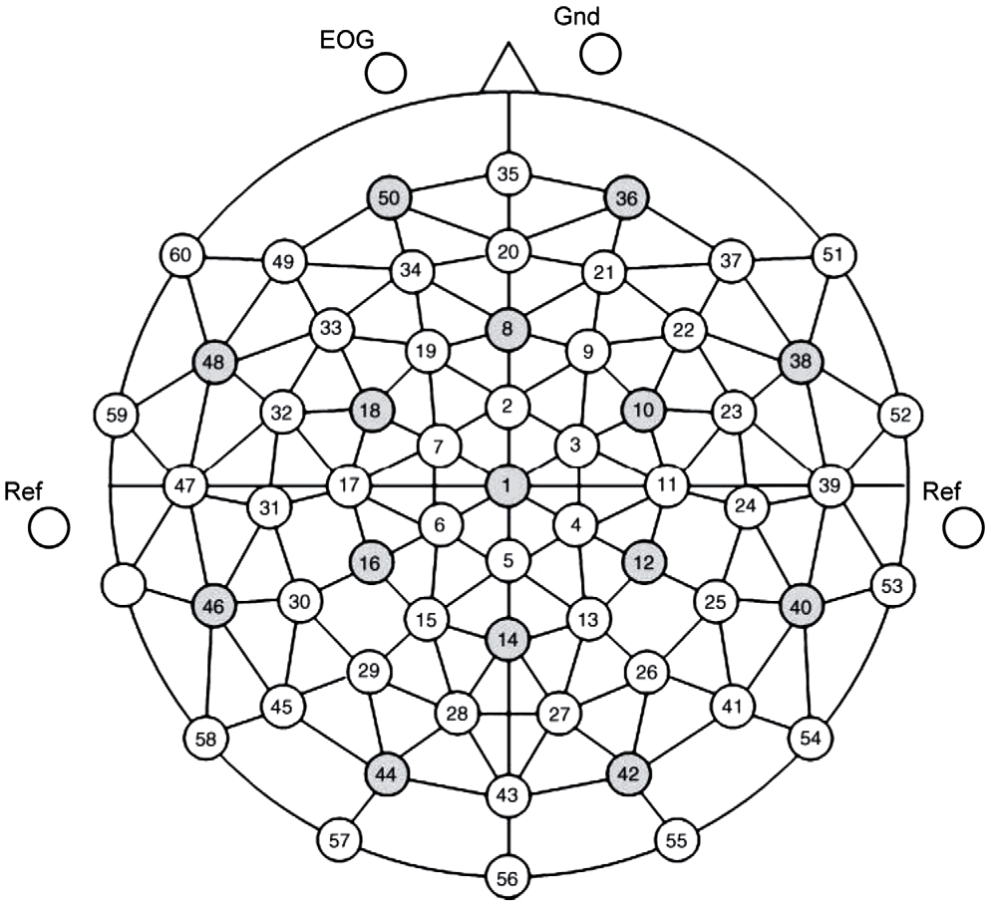
The equidistant electrode montage.

Continuous EEG was registered using a 64 channel ActiCap system (Brain Products GmbH) filtered at 0.2–200 Hz and sampled at 500 Hz with the BrainVision Recorder Professional software (Brain Products GmbH). An equidistant electrode cap was used to position 60 electrodes on the scalp (Figure 1). EEG data were recorded against the reference at the right mastoid; an additional electrode measured the voltage on the left mastoid, and the data were offline converted to a linked-mastoids reference. Bipolar EOG was computed using electrodes that were placed horizontally and vertically around the eyes. The continuously recorded data were divided into epochs of one second starting 300 ms before stimulus onset. Trials containing eye artifacts or voltage variations at any electrode above 150 µV were rejected. The signal was filtered with a pass band of 1–30 Hz. Only relevant stimuli – of semantic categories *animals* and *tools* - were selected for subsequent analysis. Differences in the number of trials between the two classes after artifact rejection did not exceed 1.5%. All offline data processing was performed using MATLAB R2008 (The MathWorks, Inc., Natic, MA) and FieldTrip, an open source Matlab toolbox for the analysis of EEG and MEG data that has been developed at our centre (http://www.ru.nl/neuroimaging/fieldtrip/).

### Optimization of the analysis

To optimize the analysis procedure, we experimented with several analysis methods. However, to ensure that tuning the procedure to a specific set of subjects did not bias our results, we used data from four subjects (the pilot group) to optimize the procedure. The pilot data was used to examine the effect of artifact removal and to set the optimal filtering parameters, as described above. Furthermore, the pilot data was used to select the optimal feature selection and classification procedure. The pilot subjects were excluded from the reported analysis.

As input to the classifier we used the time-domain representation of the event-related potentials, the voltage measurements in sixty channels over the samples at each two milliseconds, at the 0–700 ms interval after stimulus onset. The signal over all trials was standardized to have zero mean and a standard deviation of one. Bayesian logistic regression with a multivariate Laplace prior was chosen as the classifier for subsequent analysis since it has been shown to give rise to interpretable importance maps [30]. The Supporting Information (Text S1, Figure S2 and Figure S3) may be consulted for details of the employed computational method. Once this optimal analysis scheme had been developed, the remaining group of subjects (N = 20) was analyzed blindly.

### Classification procedure

Classifiers were trained to identify in single trials which of the two semantic categories (*animal* or *tool*) were presented to the subject. We imposed constraints on the obtained models that coupled parameters located closely together in time through the use of a multivariate Laplace prior (details are mentioned in Text S1). This effectively induces an adaptive temporal smoothing of the variance of estimated regression coefficients, which facilitates interpretation of the results [30]. Classification accuracy (proportion of correctly classified trials) was used to evaluate classifier performance. Since we presented equal fractions of the two categories, chance level performance was at 50%. Significance of the classification outcome was computed using a binomial test, which compares the performance of the trained classifier with that of a baseline classifier that assigns all trials to the most prevalent class [39]. The significance level was Bonferroni corrected for the number of used subjects.

The classification approach was used to conduct three different analyses. In the first analysis, only those trials corresponding to the presentation of a particular modality were used as input to the classifier and the task was to predict semantic category from EEG data. For each subject, a stratified five-fold cross-validation was performed in which the dataset was partitioned into five random subsets. Each subset was retained as the validation data for testing the model and the remaining four subsets were used as training data for that run. This process was repeated five times. The results from all the five runs, or so-called folds, were averaged to produce a single estimate of classification accuracy.

This procedure was applied a) to the entire interval of 0–700 ms post stimulus onset, and b) repeated again for small intervals of 40 ms (16 intervals from 0 up to 640 ms), in order to identify independent important data features for each time interval.

In the experimental design, we used a small set of exemplars, which were presented repeatedly throughout a session. This allowed us to match them for the linguistic characteristics (e.g. frequency of use, syllabic structure). At the same time this approach leads to interpretation problems, since the same exemplars presented in different trials appear both in training and test datasets in cross-validation. Therefore, the classifier might use exemplar-specific rather than category-specific data features to identify class-membership, effectively predicting exemplars instead of semantic categories within a modality. A proof that the classifier generalizes over the items from one semantic category would be the ability to correctly identify the category of a previously unseen exemplar. To this end, we conducted as a second analysis, an “unseen exemplar” test. We trained a classifier on all exemplars except one. The semantic category of the left-out exemplar was then predicted using the trained classifier. This procedure was repeated for each of the eight concepts in the set for each subject.

Finally, in order to study the generalization between instead of within modalities, we used a so-called transfer learning approach [40]. In previous analyses, the trials for each of the three modalities (visual, auditory and orthographical) were assumed to be independent. In the transfer learning setting, in contrast, parameters are estimated simultaneously for each of the datasets by introducing a coupling between datasets through the use of the multivariate Laplace prior. In this way, data features are identified which should allow trials to be classified correctly for each of the three modalities (see Text S1 for details). This analysis was conducted a) for the whole trial length of 700 ms, and b) for subsequent intervals of 40 ms from 0 to 640 ms post stimulus onset.

## Results

### ERP results

**Figure 2:**
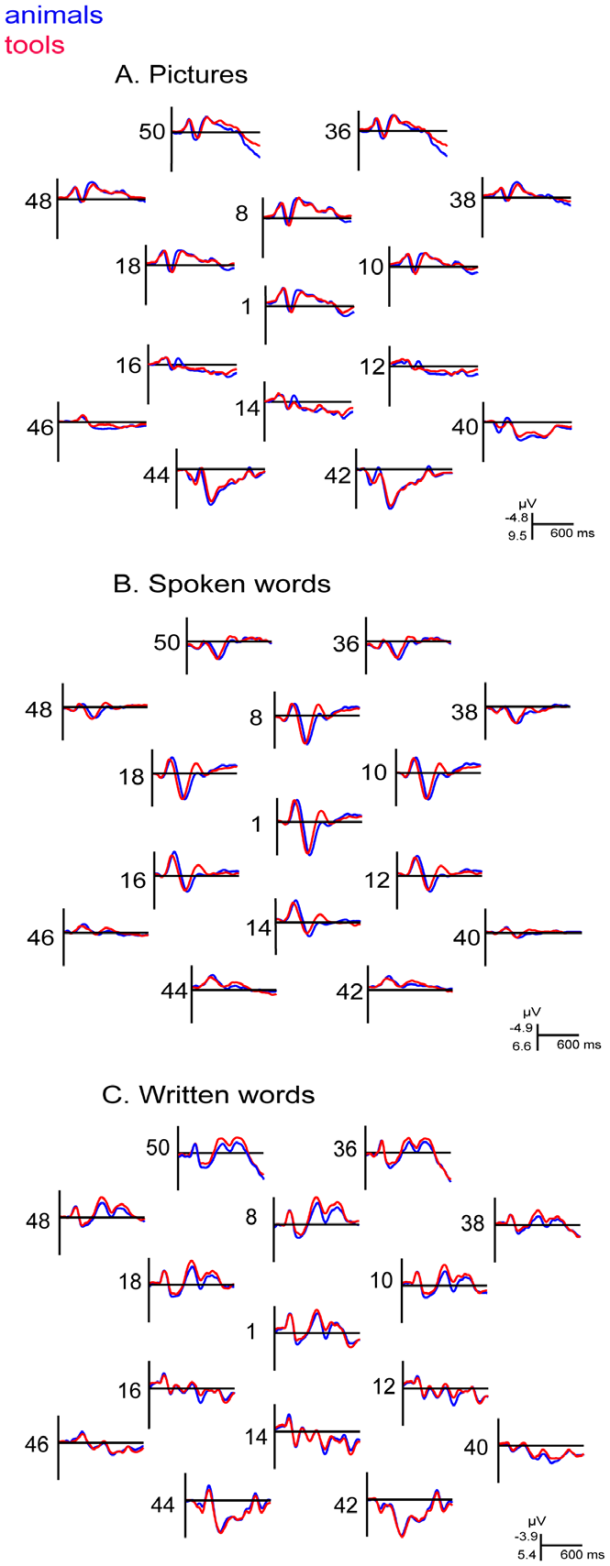
Grand average ERP results, 0–600 ms after stimulus onset. Grand average (N = 20) waveforms are shown for presentation of pictures (A), spoken words (B), and written words (C). The EEG channels are labeled according to the used electrode montage, see Figure 1.

Figure 2 shows the grand averages obtained from the entire group of twenty subjects (variability in the experimental data is illustrated in Figure S1). Inspection of the figure shows that picture presentations elicited a P1 ERP component at about 110 ms post stimulus onset followed by a visual N1 at about 160 ms post stimulus onset. These early components were largest over the posterior part of the head at infero-temporal and occipital electrodes. There were differences in the morphologies of the early components between the two categories. The P1 component in occipital electrodes peaked earlier on a response to animals and the N1 component for animals had larger amplitude at the right occipital electrodes. The early components were followed by a broad negativity that lasted from about 280 to 550 ms in fronto-central sites and occipito-temporal sites. In frontal electrodes the deflection was less negative for animals than for tools.

The spoken words elicited N1 component at centro-posterior sites peaking at 130 ms followed by P2-N2 wave at 220–310 ms. Following the N2, there was a broad negative deflection in central sites peaking between 450 and 550 ms (N400). The grand average ERP responses show that there were differences in the morphology of the P2-N2 complex in central electrodes between word categories. The N2 had larger amplitude for tools over central electrodes.

The written words elicited visual P1-N1 pattern over posterior electrodes, with larger amplitude of the N1 component over left hemisphere. At anterior sites the P1 was not clearly visible and was overlapping with a negative wave that peaked at 100 ms. In the subsequent part of the recording a broad positivity was observed in central and frontal regions, followed by frontal negativity peaking at about 300 ms [41] and subsequently by the N400 at 400–550 ms. Over parietal and centro-parietal sites there was an additional negative deflection peaking at 200 ms (N2) [42]. The N300 component at fronto-central electrodes and the subsequent N400 were less negative for animals than for tools.

### Within modalities classification results

**Figure 3:**
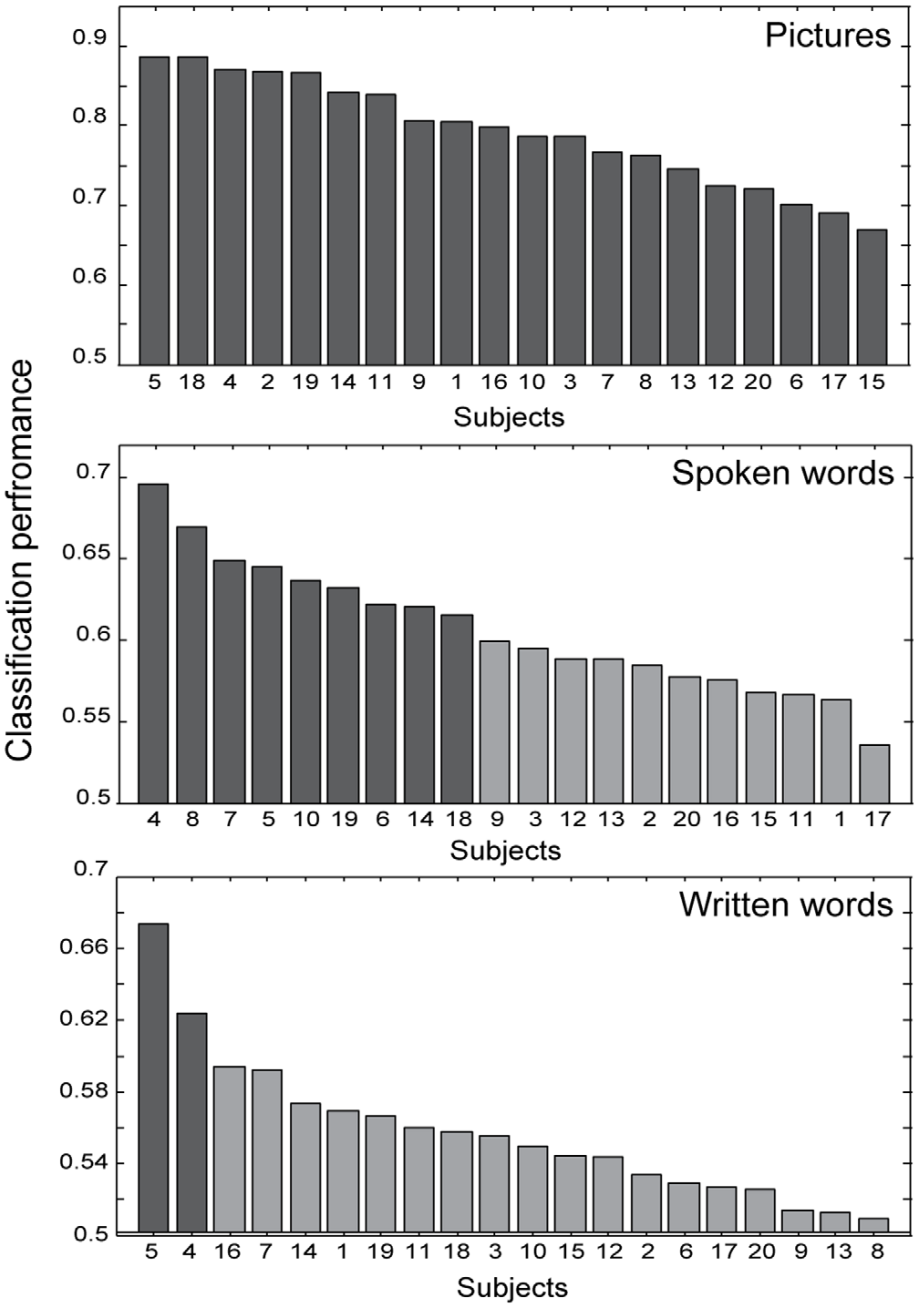
Classification performance for the three modalities. Dark bars indicate significant outcomes.

In the first analysis we trained and tested the classifiers within each of the individual modalities separately. We found strong differences in accuracies obtained for pictures in comparison with the auditory and orthographic modalities (Figure 3). For pictures, the highest classification accuracy reached over all subjects was 0.89, and classification was significant (p<0.05, Bonferroni corrected) for all twenty subjects with a mean value of 0.79 (SD = 0.07). The classifier for the auditory modality performed significantly better than chance in eight out of twenty subjects, the mean value over twenty subjects was 0.61 (SD = 0.04). The classifier for the orthographic modality performed significantly better than chance in two out of twenty subjects, with a mean group value of 0.56 (SD = 0.04).

Decoding performance did not correlate with subjects' age or task performance. Every subject performed well on the experimental task. On average they responded to 99% (SD = 1.7%) of the target stimuli.

### Important data features

**Figure 4:**
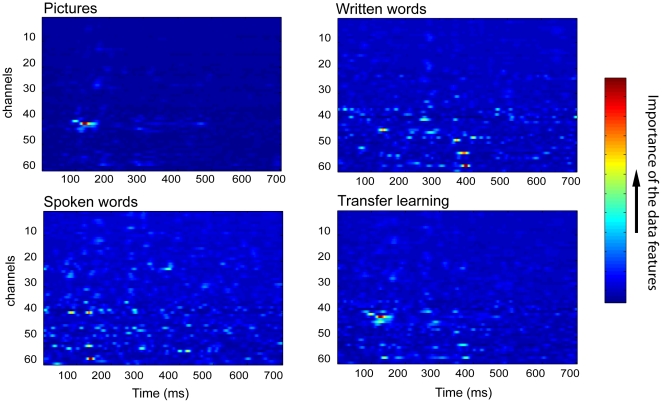
An example of the importance values for the time-channel pairs in one representative subject. Importance maps are shown for presentation of pictures (upper left panel), spoken words (bottom left panel), written words (upper right panel), and for the transfer learning (bottom right panel). The relative importance of the data features is expressed in terms of the variance of the auxiliary variables (see Text S1). Importance values are shown over time (*x*-axis, sampled each 2 ms) for 60 EEG channels (*y*-axis).

In this study we demonstrated that Bayesian logistic regression allows identification of task relevant time-channel locations from the ERPs at the single trial level in single subjects. An example of the classification model obtained for a representative subject for the entire trial duration of 700 ms is shown in Figure 4. The figure suggests that only a few channels contribute to the decoding performance. Note, however, that this sparseness is not only induced by the data but also by the employed multivariate Laplace prior. This phenomenon is described in greater detail in Text S1, see also Figure S3.

**Figure 5:**
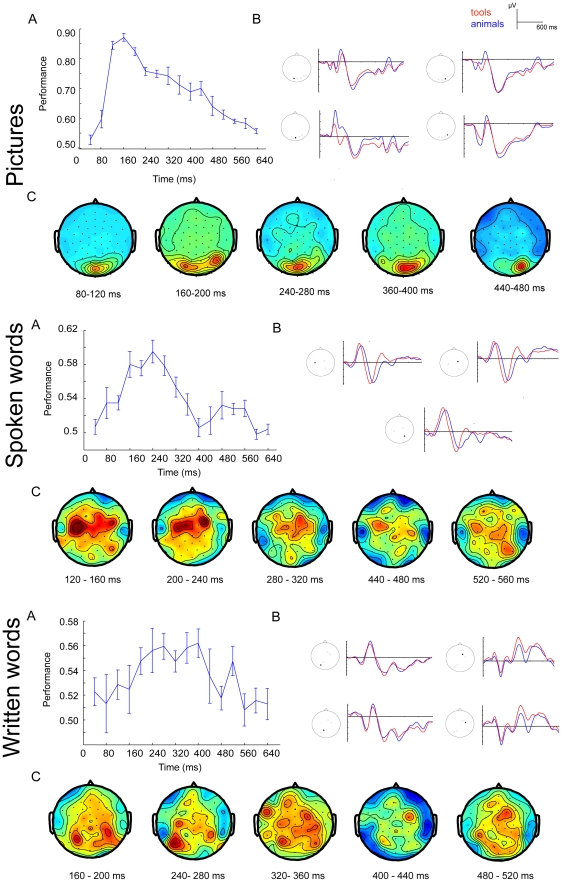
Data features important for the classification in three modalities. Upper panel – pictures, middle panel – spoken words, bottom panel – written words. A) Classification performance at 40 ms time intervals from 0–640 ms after stimulus onset. Chance level performance is at 0.50. B) The ERP waveforms from the channels that contributed most to the classification. C) The locations of the important data features in the different time intervals, starting at stimulus onset.

In order to further investigate the discriminative characteristics of ERPs on different post-stimulus latencies, we divided the trial into short time segments and conducted the classification on these segments independently. Here we present the importance maps for animal/tool classification in all three modalities (pictures, spoken and written words), averaged over five subjects that showed highest classification performance in each modality (Figure 5). Supporting material for this article includes the time course of importance maps for the twenty subjects for the three modalities (see Videos S1, S2, and S3).

#### Pictures

The classifier could reliably distinguish the category 100 ms after stimulus onset. The important data features were localized in central parieto-occipital sites 43 and 44 (POz, PO3). These results are in line with the increasing number of studies that report on early effects of visual object category in posterior locations [33], [35], [43], [44]. Later in the time interval, from 100–200 ms after stimulus onset, the focus spreads more laterally. The data features of highest importance in posterior sites correspond to the N1-P2 waveform complex of the respective ERP. In previous studies that have used univariate analysis, the time window of the N1 component has indeed been shown to be sensitive to object category. Particularly, the N1 amplitude is consistently larger for natural than for artifactual categories [34], [45], as is confirmed by our results. The N1 is traditionally thought to reflect perceptual processing. However its amplitude and latency are modulated by the experimental task, i.e. attendance to the target stimulus and categorization demand [46]–[49]. This indicates that the N1 reflects stimulus discrimination and is influenced by top-down mechanisms [50], [51]. At later latencies that are usually assumed to include semantic and associative processing, data features in the right occipital site PO4 were important for distinguishing the semantic categories.

#### Spoken words

The category of spoken words could be identified starting at 150 ms after stimulus onset, with the relevant data features located at central and fronto-central electrodes sites 9, 10, 17, and 18 (C1, C3, FC3, FC4, C4). Early important data features are left-lateralized and correspond to the N1 component, which is known to reflect the conscious detection of discrete changes in the auditory environment [52] and is also modulated by attention [53], [54]. Categories could be distinguished most easily at around 200–240 ms after stimulus onset, the interval that corresponds to the N2-P2 complex in the centro-parietal site 26 (CP4-P5), which has previously been shown to be sensitive to detection of semantic manipulations in single word listening [31], [55], [56]. Semantic categories could also be identified in late (>400 ms) ERP components, but the data features are more spread out across the scalp.

#### Written words

For written words the important data features arise around 250–400 ms after stimulus onset, focused at the left parietal electrodes 29–45 (P3–P5), with a main peak around 240–280 ms after stimulus onset. According to recent findings, recognition of written words occurs as early as 200–250 ms after stimulus onset [57]–[61]. Some previous studies also showed a strong effect of the semantic category of nouns in this time window [36]. The left occipito-temporal localization of the data features might point towards a source in left fusiform gyrus, an area that is consistently engaged in reading and particularly in written word recognition [42], [57], [62], [63]. In later latencies the classification focus spreads to the right central and centro-frontal locations 9, 10, and 12 (C4-FC4). The electrophysiological activity in these sites allowed classification at around 500 ms after stimulus onset. Late ERP waves such as the N400 and the Late Positive Component have been shown to be sensitive to semantic category in visual word presentation [32], [36].

### Unseen exemplar analysis

**Figure 6:**
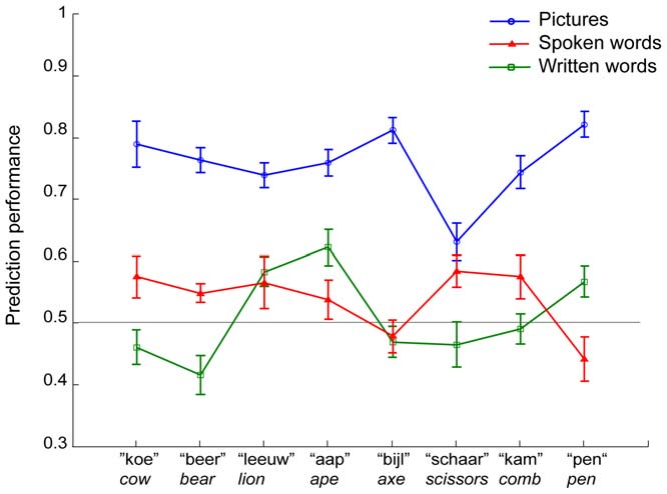
Classification performance for unseen exemplars. Chance level performance is at 0.50. English translations of Dutch stimuli are given in italics.

In the unseen exemplar analysis, our aim was to determine whether the semantic category could be predicted for previously unseen exemplars. The classifier could only solve this task reliably in the visual modality (mean accuracy 0.77, SD = 0.08, p<0.05, Bonferroni corrected, for all subjects). The classification results were non-significant for the other modalities (Figure 6).

### Classification between modalities

**Figure 7:**
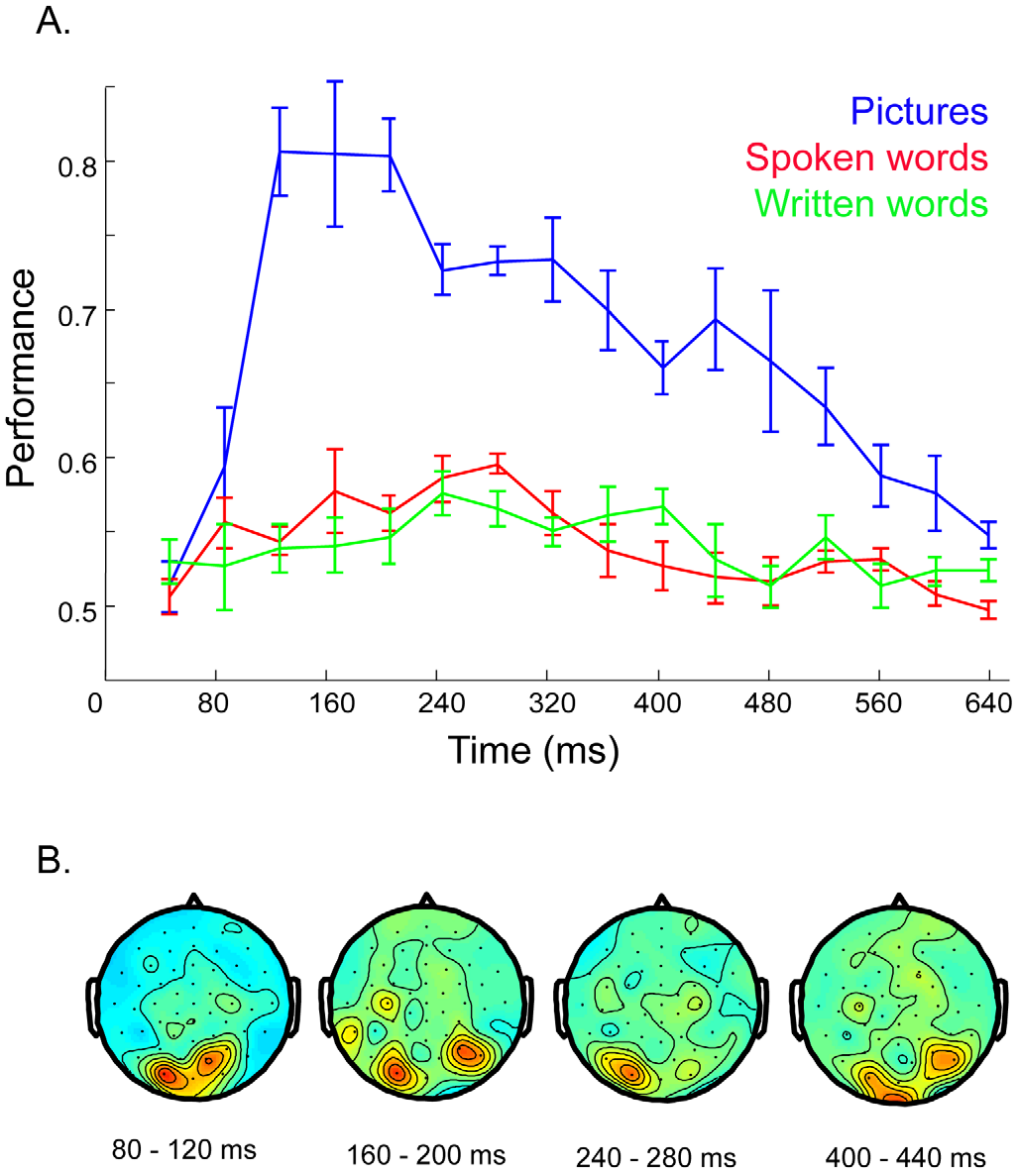
Results of the transfer learning test. A) Classification performance at 40 ms time intervals, from 0–640 ms after stimulus onset, for all three modalities. B) The locations of the important data features in the different time intervals, starting at stimulus onset. Note the strong preference for occipital areas.

To reveal the common category-related patterns across modalities we used a transfer learning approach that identified the data features that are relevant to all modalities. For this analysis we selected a subset of four subjects that showed high classification accuracies in all the modalities (subjects nr 4, 5, 7, 14). The classifier can be thought of as building a common model for the three datasets (the data from all three modalities) in each subject. The mean classification accuracy on the entire trial up to 700 ms after stimulus onset was 0.83 (SD = 0.06) for pictures, 0.66 (SD = 0.02) for audio and 0.62 (SD = 0.03) for text, see Table 2. The important data features were located in posterior sites 43 and 44 (POz, PO3); see Figure 7. The classification on short time segments revealed similar results.

**Table 2:** The results of transfer learning.

	Pictures	Spoken words	Written words
Performance	0.83	0.66	0.61
SD	0.05	0.04	0.03
Significance*	0	1.7×10^−5^ (0.001)	0.02 (0.32)

*p-values, and Bonferroni corrected p-values.

## Discussion

In this study we set out to decode the semantic category of objects from event-related EEG. We repeatedly presented a set of eight instances from two different semantic categories (*animals* and *tools*) where each instance appeared in three modalities: as a picture, as a spoken word or as a written word. Since Bayesian logistic regression is well suited for quantifying the relative importance of the data features and therefore facilitated interpretation of the obtained importance maps [30], it was the method of choice for the analysis. The distribution of electrophysiological features that contribute to the animal/tool classification in the three modalities agrees with a number of existing studies on the temporal and spatial organization of the neuronal activity underlying lexical access. In all the modalities the classifier mostly relies on early electrophysiological patterns. In addition there is a contribution from late activity in the N400 time window that is traditionally associated with semantic processes [64]. The results are highly consistent over subjects and reveal distinct brain regions and temporal structure for task-related activation.

### Classification in the visual modality

The visual modality demonstrated a clear differential response to the semantic categories. Classification performance was highly significant for all subjects. Moreover, the classifier that had been trained to discriminate *animals* and *tools* could accurately identify the category of a previously unseen exemplar from one of these categories.

The topographical distribution of the data features important for classification indicates that differential activity first takes place at centro-occipital sites and then moves laterally towards occipito-temporal locations. A large number of neuroimaging studies have reported on consistent topographical biases in the visual processing stream for pictures of animals compared with non-living objects resulting in category-specific patterns in occipito-temporal cortex (for recent reviews see [7], [8], [65]). For instance, in an fMRI study by Chao, Haxby and Martin [13], the lateral fusiform gyrus showed differential neural response to living things, whereas nonliving things elicited differential responses in the medial fusiform gyrus. Our results seem to indicate differential activation at similar locations. These findings sit naturally with the distributed domain-specific hypothesis by Caramazza [4], [6], [24], which claims that visual response is topographically segregated by semantic category. In line with this suggestion, a number of recent behavioural studies showed that category can be accessed rapidly when objects are visually presented [49], [66]–[69]. For example, in processing visual scenes, human participants can reliably make saccades to the side containing an animal in as little as 120 ms [70], and in a visual monitoring task, humans tend to detect changes concerning animals both faster and more accurately than vehicles, buildings, plants and tools [71]. These functional advantages in visual identification of animals compared to other categories could result from a segregated recognition mechanism, which evolved due to the high biological relevance of this category.

Obviously, the current results might also be explained without invoking the notion of semantic categorization on the level of visual processing. The differential activity in occipital and occipito-temporal sites could result from selectivity to certain visual attributes that happen to be more characteristic of one category than another [22], [72]. Depicted animals tend to have rounded shapes and curved lines as opposed to elongated shapes and straight lines for tools. It was recently demonstrated that if two classes of visual stimuli have a low amount of within-class variation, it is possible to get reliable classification performance using just the outputs of primary visual areas [73].

Even though the present study remains inconclusive about whether category membership or visual properties drive the classification, the present data shows that object category can be successfully decoded from the early visual components of scalp EEG. This finding is of relevance to brain-computer interface (BCI) research [74]. For instance, many studies have shown that similar patterns of brain activity arise when perceiving and imagining objects [75]–[79]. If the semantic category can be predicted for imagined concepts using EEG then this could be used for communication and control in BCI applications.

### Classification of written and spoken words

In contrast to pictures, classification of spoken and written words turned out to be more difficult. There were two main complexities. First, classification performance across subjects was lower for audio trials than for pictures, and considerably lower for text, where it was significant only for two out of twenty subjects. Second, for these modalities, the classifier failed to predict the semantic category of a previously unseen item, suggesting that the classifier could not distinguish the semantic classes, but only the exemplars, possibly through the use of perceptual differences between the exemplars. Note further that, in contrast to pictures, for spoken or written words there are no perceptual differences that are characteristic of one or the other semantic class.

According to recent psycholinguistic studies, verbal input is processed at different levels of analysis, where situation and task demands modulate the depth of semantic processing [80], [81]. This implies that the words might not necessarily have been processed at the required level. We assume this could happen in our experiment, as the experimental task did not demand the retrieval of associative-semantic knowledge. These considerations might explain poor performance for the verbal presentations of concepts. Moreover, in our experiment the stimuli were repeated many times, and it has been shown previously that category-related effects reduce with repeated stimuli [45], [82]. These issues should be taken into account in the design of future semantic encoding experiments.

### In search for amodal semantic representations

An interesting prospect when studying the semantic system is the identification of common activity patterns across different input modalities. During the initial stages of processing, the percepts of different modalities are analyzed in their respective sensory systems. Subsequently, perceptual processing, structural encoding and identification are followed by semantic-associative integration [83]. The same semantic knowledge can be accessed by various written or spoken symbols, or real world cues, so the integration stage is assumed to be modality-independent.

The transfer learning approach allowed us to obtain reasonable classification accuracies for all three modalities. A number of previous neuroimaging studies investigating amodal semantic processing found overlapping activation for pictures and words, implicating a distributed, left-lateralized neural network in frontal, peri-sylvian temporal and parietal areas [84]–[87] (for a review see [83]). However, in our study, the important electrophysiological patterns for the cross-modal classification were largely located in occipital cortical sites. It is likely that picture trials biased the classification algorithm such that mostly data features in occipital sites were selected, allowing a reliable performance for pictures and a moderate performance for the other modalities.

One possible explanation for the inability to reveal amodal semantics-related patterns with the employed procedure is due to timing differences. Auditory stimuli are spread out in time, whereas the others are presented instantaneously. Besides the differences in timing, there might be a mismatch between the electrophysiological correlates of semantic retrieval from different modalities due to the flexibility of conceptual representations. It has been recently suggested that concepts are flexibly tailored to the current contextual constraints and the access to conceptual knowledge can be modulated by focusing on certain conceptual attributes [19], [45], [88]. For example, visually related attributes are predominantly recruited in contexts that focus on the visual appearance of objects [19]. Hence, the electrophysiological signals of interest might vary too much across modalities in order to be generalizable.

### Concluding remarks

Summarizing, in this study we employed a novel multivariate approach for the analysis of semantic category-related electrophysiological brain activity. The method allows identification of the data features that are important for classification, thereby tracking down task-related activations at the single trial level in individual subjects. We showed an ability to decode the category of presented objects from the single-trial ERP waveforms. At present, the conducted experiments do not allow us to differentiate between the perceptual versus semantic origin of the obtained classification performance, so this issue remains inconclusive. Further research on the nature of semantic representations is warranted in order to be able to characterize the interactions between perceptual and conceptual processes, and how and when perceptual input transforms into conceptual representation.

## Supporting Information

Figure S1 An illustration of variability in the ERP dataset. Left panel: pictures, middle panel: spoken words, right panel: written words. A) Between-subjects variability as shown by the ERPs from 20 subjects in response to stimuli of the different modalities. This variability is quite typical for ERP experiments and is caused, amongst others, by the large variability in cortical folding between zsubjects. The single-trial analysis is challenged by the low signal-to-noise ratio of ERPs in relation to the ongoing EEG and artifacts (line noise, muscle and ocular artifacts), so it can be extremely sensitive to the individual voltage distributions. This might explain the differences in classification performance across subjects. B) An example of within-subjects trial-to-trial variability for one representative subject. The black line and blue area represent the mean and standard deviation of the electrical potentials elicited by animals over the course of the experimental session. The number of trials used for averaging: for pictures N = 285, for spoken names N = 297, for written names N = 273. The red line and aquamarine area represent the mean and standard deviation of the electrical potentials elicited by tools: pictures (N = 305), spoken names (N = 298), written names (N = 281).
(1.33 MB TIF)

Figure S2 An example of a precision matrix. The multivariate Laplace prior employed during classification is specified in terms of a precision matrix. In this example, we show the (scaled) precision matrix for five channels and ten time points where consecutive time points are coupled with a coupling strength of one hundred. The regularization parameter was set to one.
(0.31 MB TIF)

Figure S3 Tradeoff between sparseness and smoothness of the importance map. A) Correlations of the event-related responses among sixty EEG channels in one representative subject. The matrix shows correlation coefficients among the channels, for the averaged ERP in response to spoken words, 0–700 ms after stimulus onset. The electrode positions are according to the equidistant montage; see Figure 1. B) Relative importance of the data features for time-channels pairs. The importance maps demonstrate effect of no coupling (upper-left map), coupling between neighbouring channels (bottom-left map), coupling between neighbouring time-points (upper right), and coupling between both time-points and channels (bottom right); see Text S1 for details. Results from one representative subject (same as on the Panel A), for presentation of spoken words are shown. Importance values are shown over time (x-axis, sampled each 2 ms) for 60 EEG channels (y-axis).
(3.10 MB TIF)

Text S1 Details of the computational method.
(0.09 MB DOC)

Video S1 Features importance maps over 0–640 ms after the stimulus onset, 20 subjects, pictures presentations.
(7.84 MB MPG)

Video S2 Features importance maps over 0–640 ms after the stimulus onset, 20 subjects, spoken-word presentations.
(7.88 MB MPG)

Video S3 Features importance maps over 0–640 ms after the stimulus onset, 20 subjects, written-word presentations.
(7.91 MB MPG)
