# Supplementary figures and images for: Identifying Object Categories from Event-Related EEG: Toward Decoding of Conceptual Representations
Source: PLoS One. 2010 Dec 30;5(12):e14465. doi: 10.1371/journal.pone.0014465 (PMC3012689; doi:10.1371/journal.pone.0014465)

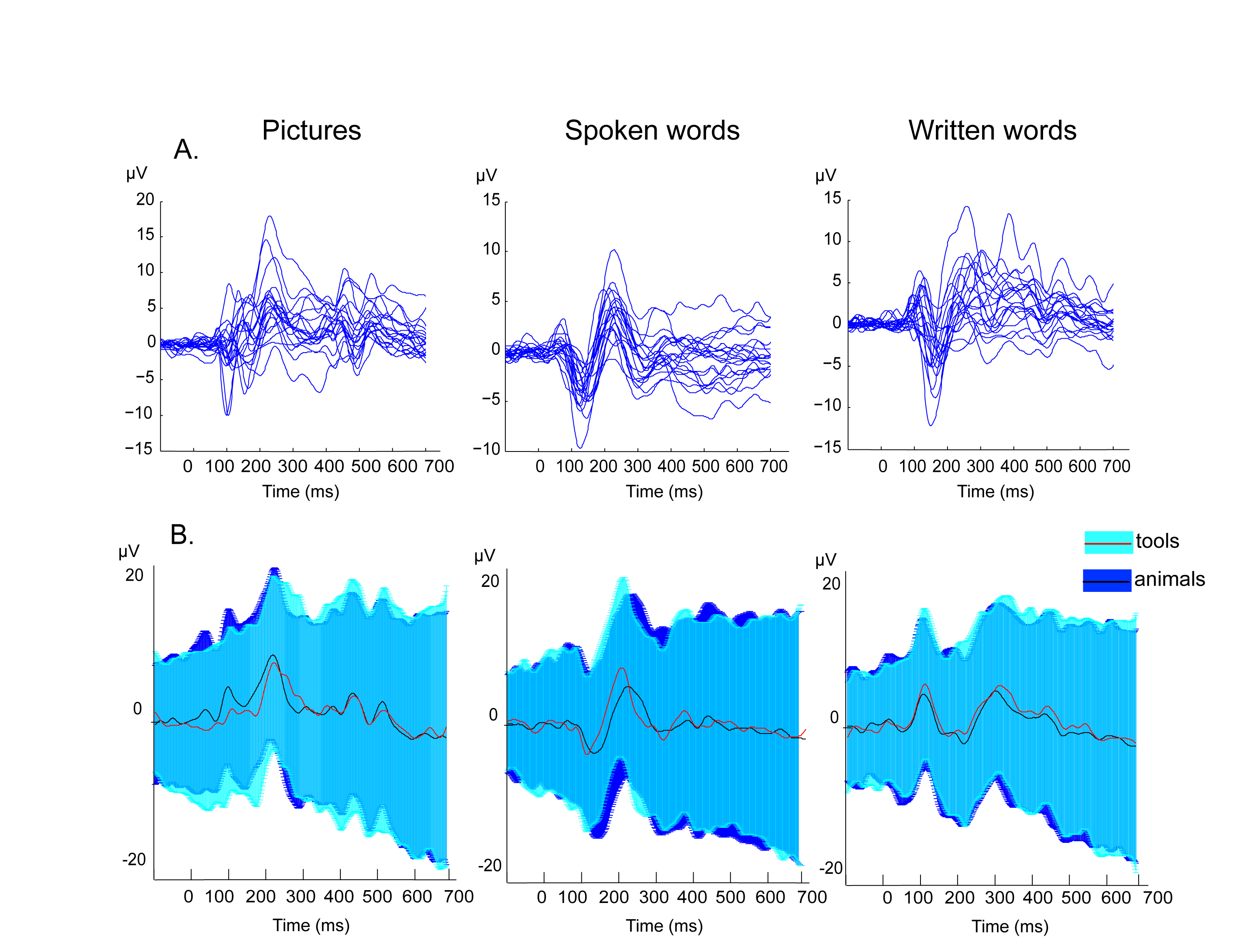

Supplement: Figure S1 — An illustration of variability in the ERP dataset. Left panel: pictures, middle panel: spoken words, right panel: written words. A) Between-subjects variability as shown by the ERPs from 20 subjects in response to stimuli of the different modalities. This variability is quite typical for ERP experiments and is caused, amongst others, by the large variability in cortical folding between zsubjects. The single-trial analysis is challenged by the low signal-to-noise ratio of ERPs in relation to the ongoing EEG and artifacts (line noise, muscle and ocular artifacts), so it can be extremely sensitive to the individual voltage distributions. This might explain the differences in classification performance across subjects. B) An example of within-subjects trial-to-trial variability for one representative subject. The black line and blue area represent the mean and standard deviation of the electrical potentials elicited by animals over the course of the experimental session. The number of trials used for averaging: for pictures N = 285, for spoken names N = 297, for written names N = 273. The red line and aquamarine area represent the mean and standard deviation of the electrical potentials elicited by tools: pictures (N = 305), spoken names (N = 298), written names (N = 281). (1.33 MB TIF) [file pone.0014465.s001.tif]

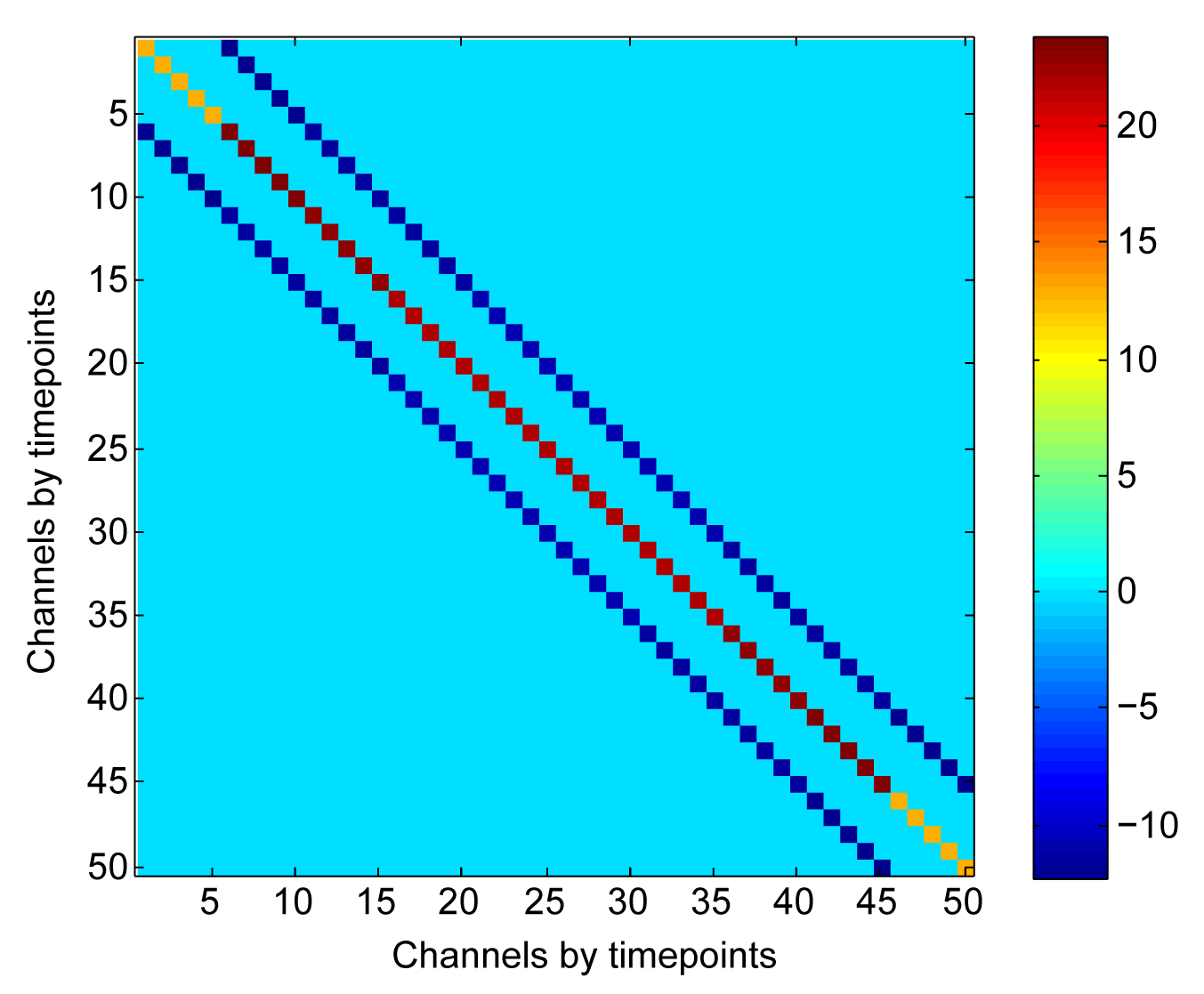

Supplement: Figure S2 — An example of a precision matrix. The multivariate Laplace prior employed during classification is specified in terms of a precision matrix. In this example, we show the (scaled) precision matrix for five channels and ten time points where consecutive time points are coupled with a coupling strength of one hundred. The regularization parameter was set to one. (0.31 MB TIF) [file pone.0014465.s002.tif]

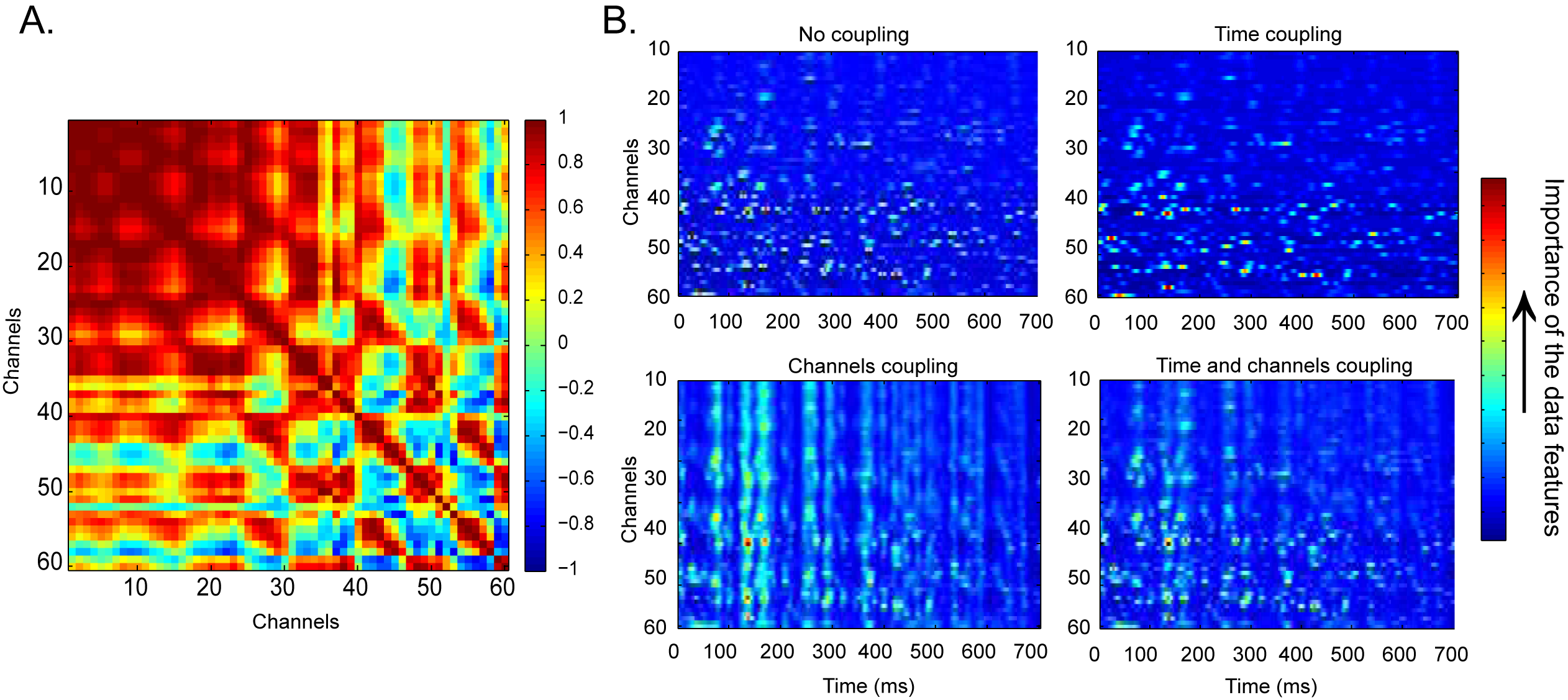

Supplement: Figure S3 — Tradeoff between sparseness and smoothness of the importance map. A) Correlations of the event-related responses among sixty EEG channels in one representative subject. The matrix shows correlation coefficients among the channels, for the averaged ERP in response to spoken words, 0–700 ms after stimulus onset. The electrode positions are according to the equidistant montage; see Figure 1. B) Relative importance of the data features for time-channels pairs. The importance maps demonstrate effect of no coupling (upper-left map), coupling between neighbouring channels (bottom-left map), coupling between neighbouring time-points (upper right), and coupling between both time-points and channels (bottom right); see Text S1 for details. Results from one representative subject (same as on the Panel A), for presentation of spoken words are shown. Importance values are shown over time (x-axis, sampled each 2 ms) for 60 EEG channels (y-axis). (3.10 MB TIF) [file pone.0014465.s003.tif]
