# Supplementary material for: Identifying Object Categories from Event-Related EEG: Toward Decoding of Conceptual Representations
Source: PLoS One. 2010 Dec 30;5(12):e14465. doi: 10.1371/journal.pone.0014465 (PMC3012689; doi:10.1371/journal.pone.0014465)
Supplement: Text S1 — Details of the computational method. (0.09 MB DOC) [file pone.0014465.s004.doc]

**Computational method**

In this paper we aim to predict the semantic category y of a presented noun from the EEG response x (the voltage measurements in 60 channels over the samples at each 2 ms), given the observed training data and hyper-parameters Θ. We use Bayesian logistic regression as the underlying classification model:

.

Using Bayes rule, we can rewrite the second term as

where is the likelihood term which does not depend on the hyper parameters Θ and is the prior to the regression coefficients. In this paper, we used a multivariate Laplace prior that can be written as a scale-mixture with auxiliary variables u and v as follows [1,2]:

,

where denotes a multivariate Gaussian with mean *μ* and covariance matrix Θ. The multivariate Laplace prior allows for controlling the prior variance of the regression coefficients through the covariance matrix Θ of the auxiliary variables u and v. We specify this covariance matrix in terms of its inverse, the precision matrix, because this facilitates the inclusion of prior knowledge about relation between covariates:

Here, θ is a scale parameter which controls regularization of the regression coefficients towards zero and R is a structure matrix where specifies a fixed coupling strength *s* between covariate *i* and covariate *j* [1,2]. V is a scaling matrix with on the diagonal, whose purpose is to ensure that the prior variance of the auxiliary variables is independent of the coupling strength. For instance, suppose we have five channels and ten time-points and we couple neighboring time-point with a coupling strength of 100. In that case, the precision matrix would be of the form shown in Supporting Figure S2.

The posterior marginals for the latent variables can be approximated using expectation propagation [3] and the posterior variance of the auxiliary variables and can be interpreted as a measure of importance of the corresponding covariate since it controls the magnitude of the regression coefficient .

In this paper, across all the discussed analyses, we coupled data features located adjacent to each other in time. EEG data is highly correlated between channels due to spatial mixing of the activity in underlying neuronal source (Supporting Figure S3, Panel A). Therefore, the differences in responses to the experimental conditions may be detected in several nearby locations. Here, in the presence of correlated features, the sparseness property of the multivariate Laplace prior emphasizes one feature, while disregarding the others. In practice, the selected data feature will be the most informative channels and time-points. The coupling of features that can be induced by the multivariate Laplace prior relaxes this property and allows strongly correlated features to be selected simultaneously. These observations are strongly related to the different regularization properties of L1 and L2 regularization, which are often employed when classification neuroimaging data.

Panel B of the Supporting Figure S3 shows the effect of inducing no coupling, coupling between neighboring channels, coupling between neighboring time-points and coupling between both time-points and channels. In practice, coupling will lead to an adaptive smoothing of the data. Classification performance typically remains relatively unaffected when varying the employed coupling, while interpretation changes due to the tradeoff between sparseness and smoothness of the importance map. In this article, we have chosen to couple neighboring time-points in order to obtain importance maps that are smooth in time and sparse in space.

In the transfer learning analysis an additional constraint was imposed. In this analysis we introduce multiple datasets for the classification, and we couple the corresponding time-channel points between the datasets. For the K datasets this is realized through an augmented data matrix

and the assumption that each covariate is coupled between datasets. I.e., the structure matrix is given by elements:

where P stands for the number of covariates between datasets. In this way, we couple covariates over datasets with coupling strength *s*.

References:

1. van Gerven MA, Cseke B, de Lange FP, Heskes T (2010) Efficient Bayesian multivariate fMRI analysis using a sparsifying spatio-temporal prior. Neuroimage 50: 150-161.
2. van Gerven M, Simanova I (2010) Concept Classification with Bayesian Multi-task Learning. In: Proceedings of the NAACL HLT 2010. pp. 10-17.
3. Minka T (2001) Expectation propagation for approximate Bayesian inference. In: Breese J, Koller D, editors. Proceedings of the Seventeenth Conference on Uncertainty in Artificial Intelligence. Morgan Kaufmann. pp. 362-369.
